# Genomic profiles and prognostic biomarkers of resectable lung adenocarcinoma with a micropapillary component

**DOI:** 10.3389/fonc.2025.1574817

**Published:** 2025-05-29

**Authors:** Jiafu Zhu, Xinhai Sun, Jiangshan Huang, Wenwei Lin, Yanyan Zhan, Junrong Yan, Yang Xu, Long Wu, Yuane Lian, Zhenyang Zhang, Jiangbo Lin

**Affiliations:** ^1^ Department of Thoracic Surgery, Fujian Institute of Thoracic and Cardiac Surgery, Fujian Medical University Union Hospital, Fuzhou, China; ^2^ Medical Department, Nanjing Geneseeq Technology Inc., Nanjing, China; ^3^ Department of Pathology, Fujian Medical University Union Hospital, Fuzhou, China

**Keywords:** lung adenocarcinoma, micropapillary component, disease-free survival, genomic features, prognostic biomarkers

## Abstract

**Background:**

Lung adenocarcinoma with a micropapillary component (LMPC) is an aggressive histologic subtype of lung cancer characterized by unique pathological features and poor prognosis. While previous studies have identified driver mutations in LMPC, its comprehensive molecular profile and prognosis-related biomarkers in the Chinese population remain poorly understood.

**Methods:**

We conducted a retrospective study of 54 stage I-III LMPC patients who underwent complete resection. Tumor samples from these patients were analyzed using broad-panel next-generation sequencing of 425 cancer-related genes. We explored the associations among clinicopathologic factors, genomic characteristics, and post-operative recurrence risk.

**Results:**

Compared to a reference cohort of 113 LADC patients, LMPC exhibited a distinct genetic profile, with a greater diversity of targetable mutations, an increased number of oncogenic pathway alterations (NPA), and more oncogenic pathway-related alterations. The mutation frequencies of *ERBB4* (11.1% vs. 1.8%, P=0.015), *BRAF* (9.3% vs. 1.8%, P=0.037), *PIK3CA* (14.8% vs. 4.4%, P=0.029), *RPTOR* (P=0.033), and *NOTCH2* (P=0.033) were significantly higher in LMPC. Additionally, LMPC patients had significantly more alterations in three oncogenic pathways (PI3K, Wnt, and TGF-β) and a significantly increased NPA (P<0.001). In stage II-III LMPC patients, *SMARCA4* mutations (13.9 months vs. not reached (NR), P=0.013) and alterations in the SWI/SNF (16.3 months vs. NR, P=0.003) and Nrf2 (17.0 months vs. NR, P=0.046) pathways were significantly associated with higher postoperative recurrence risk. Furthermore, tumor mutation burden (TMB) was significantly correlated with postoperative disease-free survival (DFS), with patients having low TMB showing prolonged median DFS compared to those with high TMB (NR vs. 16.8 months, P=0.021).

**Conclusion:**

Our study elucidates the unique genetic landscape of Chinese resectable LMPC patients and highlights high TMB and mutations in *SMARCA4*, SWI/SNF, and Nrf2 pathways as potential prognostic indicators in stage II-III disease. As these factors were not confirmed in multivariate models, they should be validated in larger, multi-center cohorts to guide future risk stratification and treatment decisions.

## Introduction

Lung cancer remains the leading cause of cancer-related mortality worldwide, with non-small-cell lung cancer (NSCLC) accounting for over 80% of all cases (1, 2). Lung adenocarcinoma (LADC) is the most prevalent NSCLC subtypes (2). The 2011 classification system proposed by the International Association for the Study of Lung Cancer (IASLC), the American Thoracic Society (ATS), and the European Respiratory Society (ERS) categorizes LADC into several histologic patterns: lepidic, acinar, papillary, solid, and micropapillary (3, 4). More recently, deep learning-based approaches have demonstrated high accuracy in classifying these LADC subtypes using whole-slide images or computed tomography (CT) images (5–7). Notably, the presence of a micropapillary component (MPC) in LADC is linked to a poor prognosis. Even a minor proportion of MPC in adenocarcinoma significantly increases the risk of disease recurrence (8–12).

Several studies have identified oncogenic mutations in LADC with a micropapillary component (LMPC), revealing a diverse range of driver mutations. For instance, a meta-analysis by Pyo and Kim reported estimated mutation rates in LMPC: 62.0% for *EGFR*, 11.8% for *KRAS*, and 10.2% for *ALK* (13). Another study involving 121 LMPC patients found *EGFR* mutations to be the most prevalent (76.9%), followed by *ALK* translocations (3.3%), *KRAS* mutations (2.5%), *HER2* mutations (1.7%), and *RET* fusions (1.7%) (14). Additionally, Ou et al. conducted both RNA-sequencing and whole-exome sequencing in 101 LMPC patients and found *MACF1*, *PCLO*, *ADGRV1*, and Fanconi Anemia pathway mutations as negative indicators for recurrence-free survival (15). Despite these insights, the molecular mechanisms underlying the poor prognosis of LMPC compared to other LADC subtypes remain poorly understood, particularly in Chinese populations. Moreover, the association between specific molecular alterations and clinical outcomes has not been comprehensively investigated. Although surgical resection remains the standard treatment for early-stage LMPC, approximately 30-50% of patients experience disease recurrence within five years postoperatively (16). Thus, identifying molecular biomarkers to stratify postoperative recurrence risk in patients diagnosed with LMPC is clinically significant, as it may inform adjuvant treatment decisions and follow-up strategies beyond conventional histopathologic staging.

In this study, we aimed to investigate the molecular characteristics and potential prognostic biomarkers in Chinese patients with resectable LMPC. We conducted a comprehensive retrospective analysis of the clinicopathological features and oncogenic mutation profiles of 54 Chinese LMPC patients who underwent surgical resection, with tumors containing at least 5% MPC. For comparison, we used an external cohort of 113 Chinese LADC patients from a previous study (17). Our research focused on comparing key genetic features and aberrant signaling pathways between LADC patients with and without MPC, and analyzing the prognostic implications of these molecular features in stage II-III LMPC patients.

## Materials and methods

### Patients and samples

We retrospectively reviewed all patients with LADC who underwent radical surgical resection at the Department of Thoracic Surgery, Fujian Medical University Union Hospital between February 2017 and December 2019. Cases with an MPC of at least 5% were initially identified through pathology reports and reviewed independently by two experienced pathologists to confirm eligibility. From this initial pool, 54 patients met all criteria for final inclusion (**Supplementary Figure S1**). Inclusion criteria were as follows: 1) Histologically confirmed LADC with ≥5% MPC. 2) Negative surgical margins (R0 resection). 3) Pathological stage I-III based on the 8th edition of the TNM staging system (18). 4) Surgical procedure was lobectomy or sublobectomy with systematic mediastinal lymph node dissection. 5) No significant cardiopulmonary comorbidities or major postoperative complications. Exclusion criteria included: 1) Death from other primary malignancies or non-cancer causes. Incomplete clinicopathological or follow-up data. 2) Prior neoadjuvant therapy. 3) NGS failure or inadequate tumor tissue for sequencing. This study received approval from the Ethics Committees of Fujian Medical University Union Hospital (No. 2019JYKY017). All patients provided written informed consent, and authors had access to information that could identify individual participants during or after data collection. Tumor tissue samples from these 54 patients were collected and prepared as formalin-fixed paraffin-embedded (FFPE) specimens. These FFPE slides were reviewed by two certified pathologists to classify the histologic subtypes of LADC according to the IASLC/ATS/ERS multidisciplinary classification system. LMPC was defined as LADC with at least 5% MPC. Based on a previous study (17), LMPC cases were further divided into MPC-low (5%≤MPC<20%) and MPC-high (MPC≥20%) groups according to the proportion of MPC content.

All patients underwent R0 resection, defined as complete tumor resection with negative margins. Surgical procedures included either lobectomy or sublobectomy, both performed with systematic mediastinal lymph node dissection. Intraoperative frozen section analysis was routinely performed on bronchial margins to confirm tumor-free status. For sublobectomy cases, an adequate surgical margin was ensured, defined as either a margin >2 cm or greater than the diameter of the tumor nodule. Systematic mediastinal lymph node dissection was defined as the complete removal of lymph nodes from at least three mediastinal stations. Specifically, for right-sided tumors, nodal stations 2R, 4R, 7, and 8 were routinely dissected, while for left-sided tumors, stations 5, 6, 7, and 8 were removed. All FFPE samples were sent to Nanjing Geneseeq Technology Inc. (Nanjing, China) for targeted next-generation sequencing (NGS) of 425 cancer-relevant genes.

Follow-up assessments were conducted every three months during the first two years after surgery, every six months in the third and fourth years, and annually thereafter. Postoperative outcomes were documented during follow-up visits and supplemented through telephone interviews. Regular evaluations included physical examinations and chest computed tomography (CT), while additional imaging modalities, such as positron emission tomography-computed tomography (PET-CT), ultrasound, endoscopy, magnetic resonance imaging (MRI), or whole-body bone scans, were utilized as clinically indicated. The follow-up period concluded at the end of June 2021, with a median follow-up duration of 30.6 months (range: 6.6-54.6 months) across all patients. Disease-free survival was defined as the interval from the date of surgery to the first occurrence of disease recurrence, cancer-related death, or the last follow-up. Local recurrence was defined as tumor recurrence within the ipsilateral chest cavity, including the surgical margin of the lung or bronchus, hilar or mediastinal lymph nodes, and malignant pleural effusion.

### DNA extraction and library construction

Genomic DNA was extracted from FFPE sections using the QIAamp DNA FFPE Tissue kit (Qiagen). For normal control, genomic DNA was extracted from white blood cells (WBCs) using the DNeasy Blood & Tissue Kit (Qiagen) to remove germline variations. DNA concentration was measured with a Qubit 3.0 fluorometer using the dsDNA HS Assay Kit (Life Technologies), and quality assessment was conducted using a Nanodrop 2000 spectrophotometer (Thermo Fisher).

Approximately 0.5-1 μg of fragmented genomic DNA from each sample was used for library preparation. Library preparation and targeted capture enrichment were performed as previously described with some modifications (19). A customized xGen lockdown probe panel encompassing 425 predefined cancer-related genes was employed for selective enrichment. The captured libraries were then amplified, purified, and quantified.

### Sequencing and bioinformatics analysis

Target-enriched libraries were sequenced on the HiSeq4000 platform (Illumina) using 2×150 bp paired-end reads. Sequencing data were demultiplexed using bcl2fastq (v2.19) and processed with Trimmomatic to remove low-quality bases (quality<15) or N bases. The cleaned data were then aligned to the hg19 reference human genome using the Burrows-Wheeler Aligner (bwa-mem). Further processing was conducted with the Picard suite (available at: https://broadinstitute.github.io/picard/) and the Genome Analysis Toolkit (GATK).

Single-nucleotide polymorphisms (SNPs) and insertions/deletions (indels) were called using VarScan2 and the HaplotypeCaller/UnifiedGenotyper in GATK. The mutant allele frequency (MAF) cutoff for single-nucleotide variants (SNVs) and indels was set at 1%. Common variants were filtered out using dbSNP and the 1000 Genomes Project. Germline mutations were excluded by comparing the results to the patient’s WBC controls.

Gene fusions were identified using FACTERA and copy number variations (CNVs) were analyzed with ADTEx. The log2 ratio cutoff for copy number gain was defined as 2.0 for tissue samples, while a log2 ratio cutoff of 0.6 was used for copy number loss. Allele-specific CNVs were analyzed by FACETS with a 0.2 drift cutoff for unstable joint segments. Tumor mutational burden (TMB) was defined as the number of nonsynonymous mutations per sample, as previously described (20). The number of oncogenic pathway alterations (NPA) was quantified as the cumulative count of somatically altered pathways per patient, encompassing ten canonical pathways profiled by The Cancer Genome Atlas (TCGA) (21) and the SWI/SNF chromatin remodeling complex pathway.

### Statistical analysis

Quantitative data are presented as the median (range) or as the number of patients (percentage). Comparisons of proportions between groups were conducted using Fisher’s exact test. Survival analysis was performed using Kaplan-Meier curves, with the P value determined by the log-rank test. Hazard ratio (HR) were calculated using the Cox proportional hazards model. Both univariate and multivariate analyses were conducted to investigate the association between various variables and disease-free survival (DFS). A two-sided P value of less than 0.05 was considered statistically significant for all tests unless otherwise specified. All analyses were conducted using R version 3.6.0.

## Results

### Patient characteristics

The clinicopathological features of the 54 Chinese patients with resectable LMPC are summarized in **Table 1**. Paired treatment-naive tumor tissue and corresponding WBCs control samples were available for each patient. The cohort consisted of 20 females (37.0%) and 34 males (63.0%), with a median age of 60 years. The study included an equal number of stage I and stage II-III patients, and 25 patients (46.3%) had a history of smoking. Based on the percentage of MPC in the tumor, 29 patients (53.7%) were classified as MPC-low (5%≤MPC<20%) and 25 patients (46.3%) as MPC-high (MPC≥20%). Lymph node metastases were observed in 18 patients (33.3%) while lymphovascular invasion and pleural invasion were detected in 6 patients (11.1%) and 8 patients (14.8%) respectively. Following surgical resection, 24 patients (44.4%) received adjuvant chemotherapy and/or adjuvant EGFR or ALK tyrosine kinase inhibitors (TKIs) treatment. Additionally, approximately 26% (7/27) of stage II-III patients received adjuvant EGFR or ALK TKIs treatment, while none of the patients in our cohort received immunotherapy. No statistically significant differences in clinicopathological features were observed between the 54 LMPC patients and the 113 reference LADC patients (**Table 1**).

**Table 1:** Clinicopathologic characteristics of LMPC patients enrolled in this study compared to the reference cohort of 113 LADC patients.

Characteristics	No. (%)		p value
	54 LMPC patients	113 reference LADC patients	
Age			0.869
<60	26 (48.1%)	57 (50.4%)	
≥50	28 (51.9%)	55 (48.7%)	
NA	0 (0%)	1 (0.9%)	
Gender			0.070
Male	34 (63.0%)	50 (44.2%)	
Female	20 (37.0%)	56 (49.6%)	
NA	0 (0%)	7 (6.2%)	
Smoking status			1.000
Never	29 (53.7%)	2 (0.9%)	
Ever	25 (46.3%)	1 (1.8%)	
NA	0 (0%)	110 (97.3%)	
TNM stage			0.244
I	27 (50.0%)	45 (39.8%)	
II-IV	27 (50.0%)	68 (39.0%)	
LMPC content			-
MPC-low	29 (53.7%)	–	
MPC-high	25 (46.3%)	–	
*EGFR* status			0.241
WT	19 (35.2%)	52 (46.0%)	
Mutant	35 (64.8%)	61 (54.0%)	
Lymph node metastasis			-
No	36 (66.7%)	–	
Yes	18 (33.3%)	–	
Pleural invasion			-
No	46 (85.2%)	–	
Yes	8 (14.8%)	–	
Intravascular tumor thrombus			-
No	48 (88.9%)	–	
Yes	6 (11.1%)	–	
Adjuvant therapy			-
No	30 (55.6%)	–	
Yes	24 (44.4%)	–	

LMPC, lung adenocarcinoma with micropapillary component; LADC, lung adenocarcinoma; No., number; WT, wild type; NA, not available.

### Driver gene characteristics of LMPC

**Figure 1:**
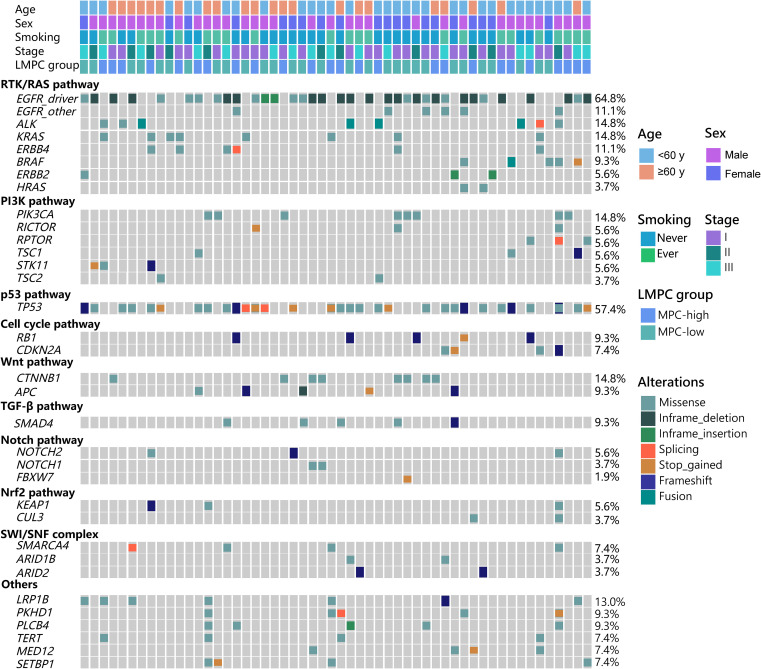
The genetic landscape of the 54 stage I-III LMPC patients enrolled in this study. The demographic and clinical features of the patients were listed at the top of the graph while various oncogenic pathway-associated mutations were listed below. LMPC, lung adenocarcinoma with micropapillary component; MPC, micropapillary component.

As shown in **Figure 1**, the most commonly mutated genes in LMPC were *EGFR* (75.9%), *TP53* (57.4%), *KRAS* (14.8%), *ALK* (14.8%), and *PIK3CA* (14.8%). Driver mutations, including *EGFR*, *ALK*, *KRAS*, *ERBB2*, *BRAF*, and *HRAS*, were detected in over 90% of patients (**Figure 1**, **Supplementary Figure S2**). *ALK* fusions were detected in 4 patients (7.4%), and *KRAS* mutations were present in 8 patients (14.8%), with 3 cases involving the *KRAS* p.G12C mutation (**Figure 1**, **Supplementary Figure S2**). RTK/RAS was the most frequently mutated signaling pathway, primarily due to the high frequency of *EGFR* mutations. Specifically, 64.8% of the patients had *EGFR* driver mutations, with an additional 11.1% harboring other *EGFR* mutations. Other RTK/RAS-related gene mutations were also prevalent, including *ALK* (14.8%), *KRAS* (14.8%), *BRAF* (9.3%), and *ERBB2* (5.6%) (**Figure 1**).

Genetic alterations in the PI3K pathway were commonly detected, with mutations in *PIK3CA* (14.8%), *RICTOR* (5.6%), *RPTOR* (5.6%), *STK11* (5.6%), *TSC1* (5.6%), and *TSC2* (3.7%). In the Notch pathway, mutation frequencies were 1.9% for *FBXW7*, 3.7% for *NOTCH1*, and 5.6% for *NOTCH2*. For the Nrf2 pathway, *KEAP1* and *CUL3* had mutation rates of 5.6% and 3.7%, respectively. Alterations were also observed in the SWI/SNF pathway, with mutations in *SMARCA4* (7.4%), *ARID1B* (3.7%), and *ARID2* (3.7%) (**Figure 1**). Overall, we observed a relatively high frequency of oncogenic driver mutations and aberrant signaling pathways in Chinese resectable LMPC patients, highlighting the complexity and heterogeneity of this disease.

### Comparison of mutation profiles between LMPC and LADC patients

**Figure 2:**
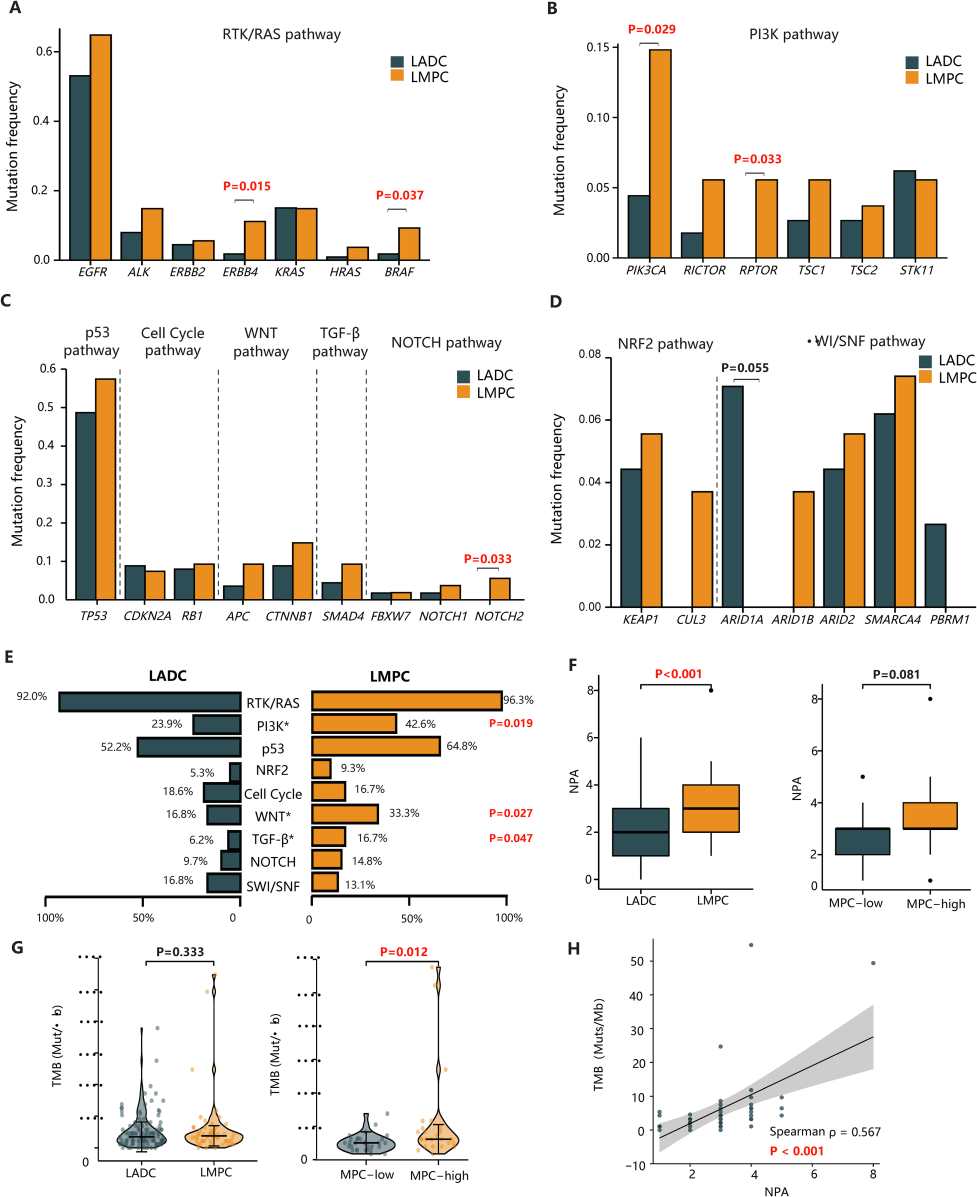
The comparison of genetic features between our LMPC patients and the reference cohort. **(A–D)** The bar plots of the mutation frequency of significantly altered genes between LMPC and LADC groups in the RTK/RAS, PI3K, p53, Cell cycle, Wnt, TGF-β, Notch, Nrf2, and SWI/SNF pathways. **(E)** The comparison of the frequency of aberrant oncogenic pathways between LMPC and LADC groups. **(F)** The comparison of NPA in tumors categorized based on the presence of MPC (LMPC vs. LADC) or the MPC content (MPC-low vs. MPC-high). **(G)** The violin plot of the differential TMB levels in tumors categorized based on the presence of MPC (LMPC vs. LADC) or the MPC content (MPC-low vs. MPC-high). **(H)** The scatterplot of TMB versus NPA in the LMPC group. The Spearman’s correlation coefficient(ρ) and the associated P value were labeled on the figure. LADC, lung adenocarcinoma; LMPC, lung adenocarcinoma with micropapillary component; MPC, micropapillary component; TMB, tumor mutation burden; NPA, the number of oncogenic pathway alterations.

Given that LMPC and the reference cohort of 113 LADC patients exhibited distinct clinical outcomes, we hypothesized that underlying molecular and genetic differences might exist between these two patient populations. Therefore, we compared the mutational characteristics of our LMPC patients with those of the 113 Chinese LADC patients described in recent literature (17). The mutation frequencies of *EGFR*, *ALK*, *ERBB2*, *KRAS*, and *HRAS* were similar between the LMPC and LADC groups. However, mutations in *ERBB4* (11.1% vs. 1.8%, P=0.015) and *BRAF* (9.3% vs. 1.8%, P=0.037) were significantly more enriched in the LMPC group (**Figure 2A**).

Beyond the RTK/RAS pathway, mutations in the PI3K pathway (*PIK3CA*: 14.8% vs. 4.4%, P=0.029; *RPTOR*: 5.6% vs. 0%, P=0.033) and the Notch pathway (*NOTCH2*: 5.6% vs. 0%, P=0.033) were more frequently observed in LMPC patients than in LADC patients (**Figures 2B, C**). In contrast, the mutation frequency of *ARID1A* (7.1% vs. 0%, P=0.055) in the SWI/SNF pathway tended to be higher in LADC than in LMPC, although this result was not statistically significant (**Figure 2D**).

We further compared differences in oncogenic pathways between the LADC and LMPC groups, analyzing nine classical oncogenic pathways altered in at least five LMPC patients (**Figure 2E**). Consistent with the gene-level mutational analysis, LMPC patients harbored more genetic alterations in several oncogenic pathways, including PI3K (42.6% vs. 23.9%, P=0.019), Wnt (33.3% vs. 16.8%, P=0.027), and TGF-β (16.7% vs. 6.2%, P=0.047). Additionally, the NPA in the LMPC group was significantly higher than in the LADC group (P<0.001) (**Figure 2F**). Similarly, the NPA in the MPC-high group tended to be higher than in the MPC-low group (P=0.081) (**Figure 2F**).

We then compared the TMB levels between LADC and LMPC patients and found no significant difference between the two groups (3.44 vs. 3.76, P=0.333) (**Figure 2G**). However, a further comparison of TMB levels between MPC-low and MPC-high patients revealed that the TMB levels were significantly higher in the MPC-high group (3.22 vs. 4.30, P=0.012) (**Figure 2G**). Moreover, there was a positive correlation between TMB and NPA in LMPC patients (Spearman’s correlation coefficient, r=0.567, P<0.001) (**Figure 2H**). Overall, LMPC patients, especially those with high MPC, tended to carry more oncogenic alterations, signaling pathway aberrations, and higher mutational burdens.

### The association between clinicopathological characteristics and prognosis in LMPC patients

**Figure 3:**
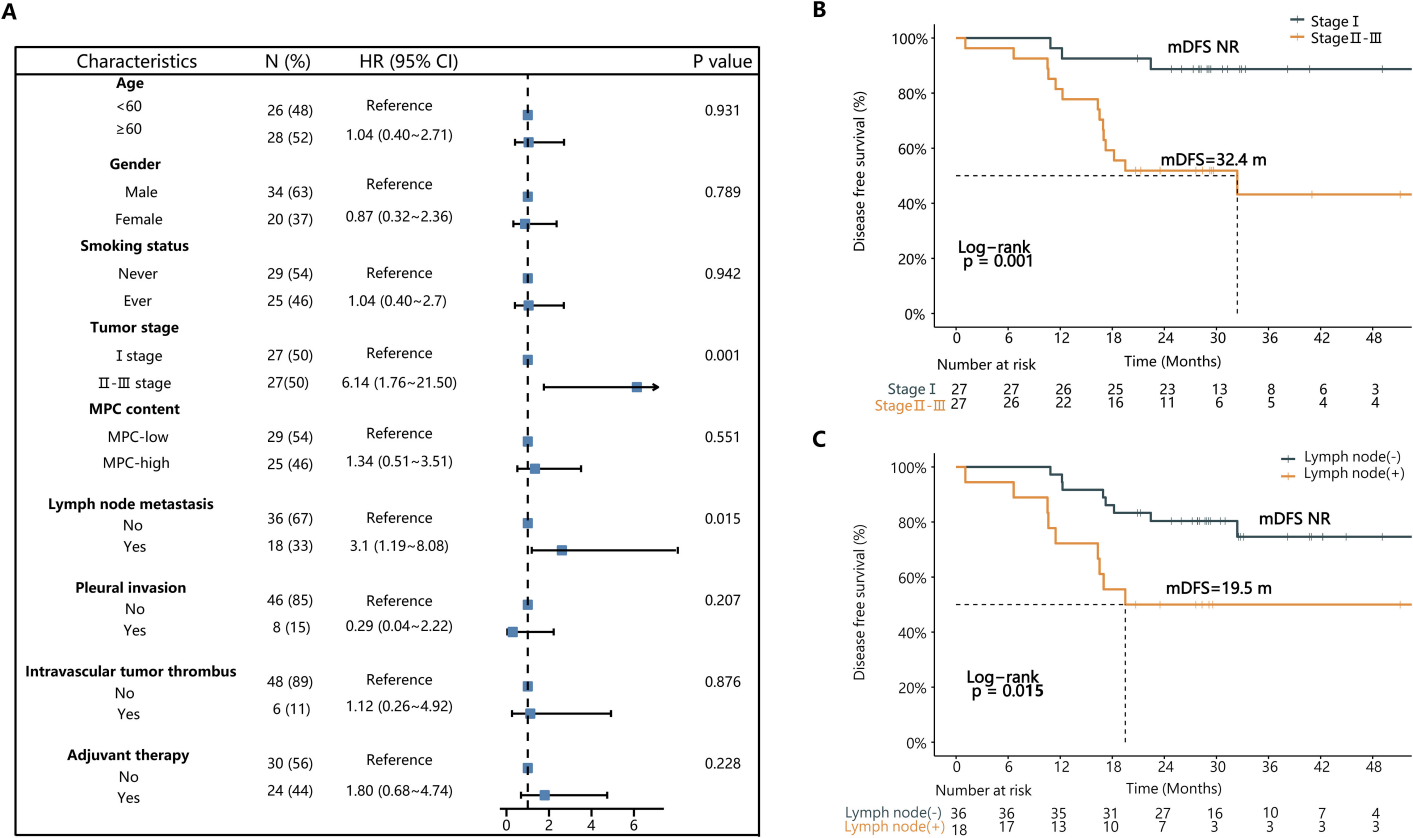
The univariate Cox regression analysis of clinicopathologic characteristics associated with DFS in the 54 patients. **(A)** The forest plot presents hazard ratios (HRs) of various clinicopathological characteristics associated with DFS. **(B)** The Kaplan-Meier curve of DFS in stage I vs stage II-III patients. **(C)** The Kaplan-Meier curve of DFS in patients with or without lymph node metastasis. MPC, micropapillary component; mDFS, median disease-free survival; NR, not reached.

As resectable LMPC patients often experience poor prognosis, we explored the association between post-surgical DFS and various clinical characteristics. Univariate Cox regression analysis indicated that clinicopathological factors such as age, sex, smoking history, MPC content, pleural invasion, lymphovascular invasion, and adjuvant therapy did not significantly affect patients’ prognosis. However, tumor stage and lymph node metastasis status were significantly correlated with postoperative DFS in LMPC patients (**Figure 3A**).

Specifically, stage I patients had significantly better postoperative median DFS (mDFS) compared to stage II and III patients (Not reached (NR) vs. NR vs. 18.2 months, P=0.004, HR = 6.14) (**Figure 3B**; **Supplementary Figure S3A**). There was no significant difference in mDFS between stage II and stage III patients (NR vs. 18.2 months, P=0.520) (**Supplementary Figure S3A**). In univariate Cox regression analysis for stage II-III LMPC patients, none of the tested clinicopathological factors had a significant effect on mDFS (**Supplementary Table S1**).

Among the 54 LMPC cases, patients without lymph node metastasis demonstrated significantly better DFS compared to those with lymph node metastasis (NR vs. 19.5 months, P=0.015, HR=3.10) (**Figure 3C**). Furthermore, no significant difference in DFS was observed between MPC-high and MPC-low patients (P=0.550), indicating that micropapillary content alone may not independently predict prognosis in this cohort (**Supplementary Figures S3B, C**).

### Correlation analysis of gene mutations, signaling pathways, TMB and recurrence in surgically resected stage II-III LMPC patients

Considering that all the investigated clinicopathological factors failed to stratify DFS in stage II-III LMPC patients (**Supplementary Table S1**), and recognizing the clinical importance of estimating prognosis in patients with more advanced diseases, we decided to explore the prognostic effects of tumor mutations and aberrant signaling pathways in these patients. Genes or pathways that were altered in ≥3 patients were included in the analysis, and their correlations with mDFS were examined.

**Figure 4:**
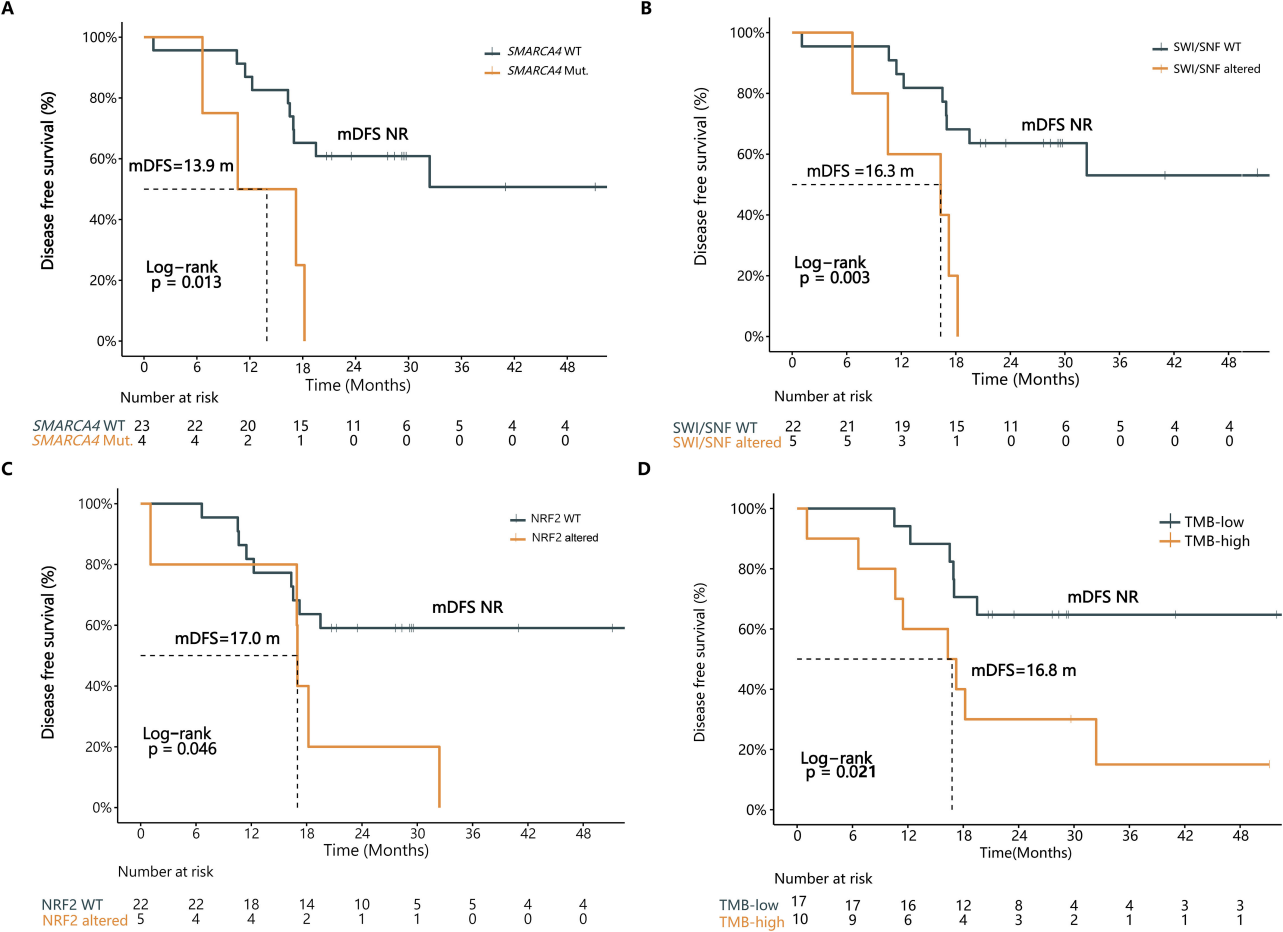
Multiple genetic features were associated with DFS in stage II-III LMPC patients. The Kaplan-Meier curve of DFS in stage II-III LMPC patients in strata of **(A)** *SMARCA4* mutation, **(B)** SWI/SNF pathway alteration, **(C)** Nrf2 pathway alteration, or **(D)** TMB status. WT, wild type; Mut., mutation; mDFS, median disease-free survival; NR, not reached; TMB, tumor mutation burden.

Among the 27 stage II-III LMPC patients, *SMARCA4* mutations were significantly associated with poorer mDFS (13.9 months vs. NR, P=0.013) (**Figure 4A**; **Supplementary Table S2**). Additionally, patients harboring *KEAP1* mutations tended to have poorer mDFS, although this difference was not statistically significant (18.2 months vs. NR, P=0.159) (**Supplementary Table S2**).

Patients with aberrations in the SWI/SNF pathway exhibited significantly shorter mDFS (16.3 months vs. NR, P=0.003) (**Figure 4B**; **Supplementary Table S2**). Similarly, patients with Nrf2 pathway-related mutations had significantly reduced mDFS (17.0 months vs. NR, P=0.046) (**Figure 4C**; **Supplementary Table S2**).

Next, we analyzed the correlation between TMB and DFS in the 27 stage II-III LMPC patients. The findings suggest that mutations in specific genes and pathways could serve as prognostic indicators in more advanced LMPC cases, providing valuable insights for clinical management and therapeutic strategies.

In this study, the highest tertile of TMB was defined as TMB-high, reflecting a TMB≥6.44 mutations per megabase (mut/Mb) in the 27 stage II-III LMPC patients. The intermediate and low tertiles were defined as TMB-low. Patients with TMB-low experienced a significant DFS benefit compared to those with TMB-high (Not reached (NR) vs. 16.8 months, P=0.021, HR=3.26) (**Figure 4D**; **Supplementary Table S2**).

Therefore, several genetic features, including specific gene mutations, pathway aberrations, and TMB, can potentially serve as postoperative prognostic biomarkers in stage II-III LMPC patients. These findings highlight the importance of molecular profiling in predicting clinical outcomes and guiding treatment decisions for patients with advanced LMPC.

### Multivariate cox regression analysis of DFS and molecular characteristics in stage II-III LMPC patients

We conducted a multivariate Cox regression analysis using the molecular features that were significantly associated with DFS in stage II-III LMPC patients. Surprisingly, none of the selected molecular features were statistically significant in the multivariate analysis, although the insignificance of the SWI/SNF pathway (P=0.147) might be attributed to the limited sample size (**Supplementary Table S3**). This implies that the SWI/SNF pathway, the Nrf2 pathway, and TMB are not independent prognostic factors in stage II-III LMPC, suggesting possible correlations among these features.

To test this hypothesis, we performed correlation analyses between various molecular features. The mutation status of multiple genes, including *TP53*, *LRP1B*, *SMARCA4*, *KEAP1*, and *PKHD1*, was significantly associated with increased TMB (2.7 vs. 4.3, P=0.023; 3.2 vs. 8.6, P=0.019; 3.2 vs. 16.6, P=0.016; 3.2 vs. 49.4, P<0.001; 3.2 vs. 37.1, P=0.011) (**Supplementary Figures S4A, B**). Additionally, patients with altered p53, Nrf2, SWI/SNF, and TGF-β pathways showed a significant or close-to-significant association with higher TMB (**Supplementary Figures S4C, D**). Overall, the alterations in the SWI/SNF and Nrf2 pathways were correlated with increased TMB, which may explain the insignificant results in the multivariate analysis. These findings indicate that while individual molecular features may not independently predict prognosis, their interactions and combined effects on TMB could play a crucial role in the clinical outcomes of stage II-III LMPC patients.

**Figure 5:**
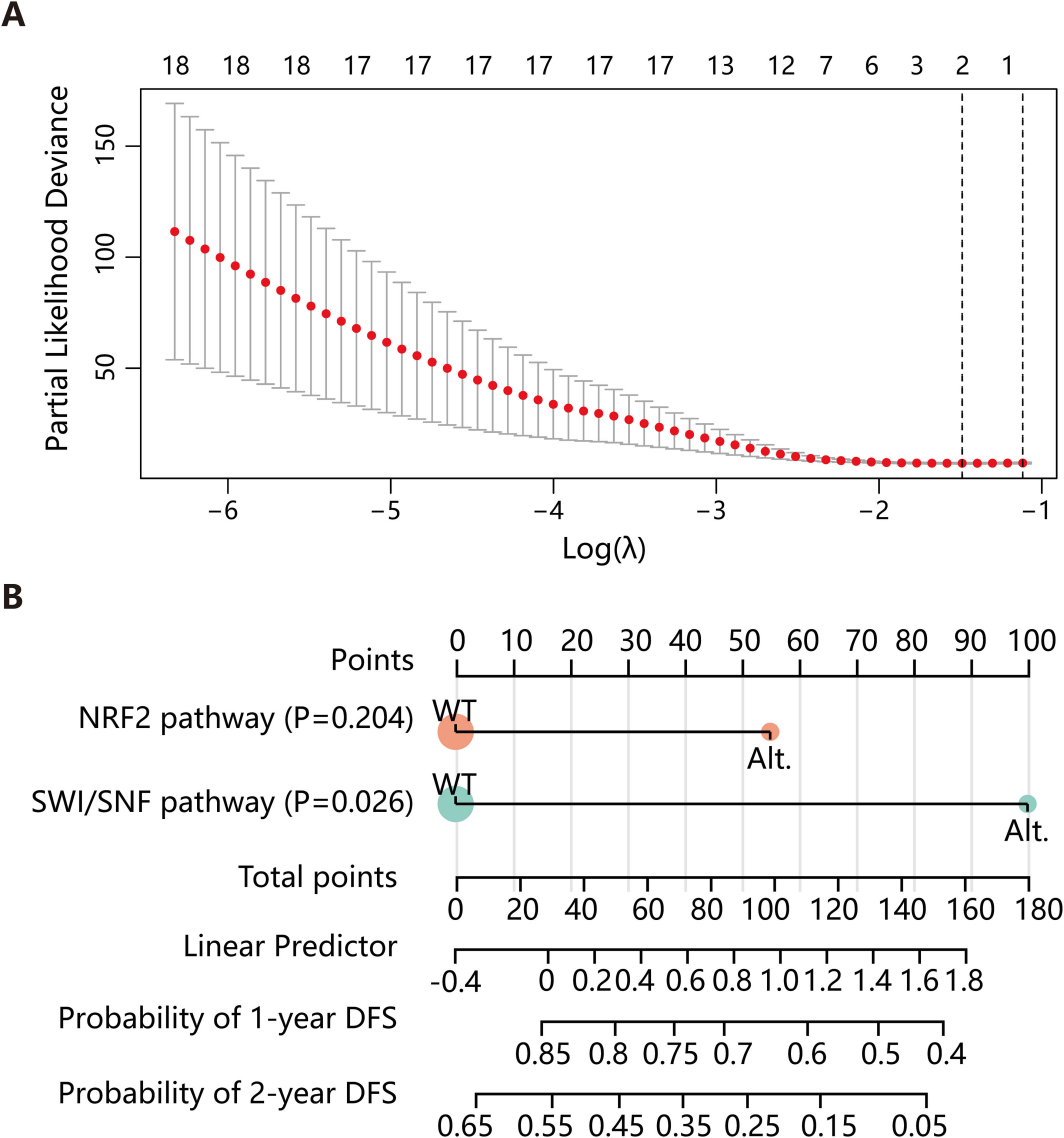
Construction of the DFS predictive model. **(A)** LASSO regression was employed to select variables for constructing the DFS prediction model. **(B)** A nomogram was developed based on a Cox proportional hazards model, incorporating the Nrf2 and SWI/SNF pathways. DFS, disease-free survival; LASSO, least absolute shrinkage and selection operator; Alt., alteration; WT, wild type.

Lastly, we conducted a preliminary prognostic modeling analysis using the 27 stage II-III LMPC patients in our cohort as a training set. We applied the least absolute shrinkage and selection operator (LASSO) regression for feature selection from a panel of over ten clinical and molecular features, including TMB, pathway alterations, and NPA classification. This analysis identified the Nrf2 and SWI/SNF pathways as the most predictive combination of features for DFS (**Figure 5A**). Subsequently, we constructed a nomogram based on a Cox proportional hazards model integrating these two molecular features. The performance of the model was evaluated using the concordance index (C-index), which was 0.792 (95% CI: 0.676-0.908, P = 7.73e-07) (**Figure 5B**), indicating good discriminative ability within this cohort.

## Discussion

LMPC, a histologic subtype of LADC, is generally associated with poor prognosis. Despite its clinical significance, the molecular characteristics and associated prognosis of LMPC have been under-investigated, particularly in the Chinese population. To address this gap, we conducted a systematic analysis to characterize the molecular and clinical features of 54 Chinese resectable LMPC patients. Our findings revealed that LMPC tumors possess a unique genetic profile, featuring more diverse targetable mutations, an increased NPA, and more extensive oncogenic pathway alterations compared to LADC tumors. Specifically, the mutational frequencies of *ERBB4*, *BRAF*, *PIK3CA*, *RPTOR*, and *NOTCH2* were significantly higher in LMPC than in LADC. LMPC patients were also more likely to harbor genetic alterations in multiple oncogenic pathways, including PI3K, Wnt, and TGF-β. Among stage II-III LMPC patients, molecular features such as *SMARCA4* mutations, SWI/SNF pathway alterations, Nrf2 pathway alterations, and TMB were significantly associated with postoperative recurrence risk.

We discovered that driver gene mutations, including *EGFR*, *KRAS*, *ALK*, *ERBB2*, and *BRAF*, occurred in 91% of LMPC tumors. Previous studies on Asian LMPC patients have shown that mutations in *EGFR*, *ALK*, and *KRAS* occur in approximately 65-75%, 4-7%, and 3-6% of cases, respectively (13, 14, 22). These rates are higher than those observed in other LADC subtypes, which is generally consistent with our results but with some discrepancies. In this study, all exon regions of the driver genes in the predefined gene panel (425 cancer-related genes) were sequenced using NGS technology. Compared to previous RT-PCR hotspot sequencing technologies that focused on a few driver mutations, NGS provides a comprehensive characterization of common and rare driver gene mutations in LMPC. For example, five patients with *CHMP3-ALK* fusion, *SND1-BRAF* fusion, and multiple driver gene mutations were identified in our LMPC cohort.

To better understand the differences in signaling pathways between LADC patients with and without an MPC, we conducted a similar analysis. The LMPC group exhibited a higher NPA than the reference Chinese LADC patients, with three oncogenic pathways (PI3K, Wnt, and TGF-β) frequently altered in LMPC. Caso et al., who performed NGS analysis on 604 LADC patients, reported that NPA and TMB were associated with increasing subtype invasiveness and significantly higher frequencies of the micropapillary or solid subtypes (23). In our study, the mutation frequencies of altered oncogenic pathways in LMPC patients were significantly higher than those in LADC patients, and a similar trend was observed when comparing MPC-high with MPC-low tumors. Although there was no significant difference in TMB between the LMPC and LADC groups, the MPC-high group had significantly higher TMB levels than the MPC-low group (P=0.012), consistent with previous reports showing that TMB tended to increase with elevated MPC percentage (17).

In our study, *SMARCA4* mutations and alterations in the SWI/SNF signaling pathway were significantly associated with poor outcomes in stage II-III LMPC patients. *SMARCA4* mutations, reported as the most frequent mutations in the SWI/SNF complex, are associated with poor prognosis in lung cancer, although *SMARCA4*-mutated lung cancer may be more sensitive to immunotherapy (24). Two other studies showed that *SMARCA4* mutations were related to significantly shorter overall survival and that the presence of *SMARCA4* mutations might lead to poorer immunotherapy outcomes in NSCLC patients with *KRAS* co-mutation (25, 26). Another SWI/SNF complex gene, *ARID1A*, has been reported to contribute to better immunotherapy outcomes (25, 27). A similar trend was observed in our LMPC cohort, although the result was not statistically significant (P=0.055). Therefore, *SMARCA4* mutation could potentially serve as a prognostic and/or predictive biomarker in NSCLC. Given that immunotherapy efficacy may not be ideal in LMPC patients with *SMARCA4* and *KRAS* co-mutations, it is worth exploring the clinical utility of testing *SMARCA4*, *KRAS*, and *ARID1A* mutations in LMPC patients to predict their eligibility for immunotherapy.

We found that genetic alterations in *KEAP1*, a key component of the Nrf2 pathway, were correlated with poor prognosis in stage II-III LMPC patients. Mutations in the Nrf2 pathway, an important regulator of redox balance and cell homeostasis, are common in NSCLC and are associated with increased tumor growth and aggressiveness (28). Recent studies suggest that *KEAP1*/*NRF2* alterations in NSCLC serve as biomarkers of poor prognosis and contribute to resistance to various cancer treatments, such as chemotherapy, radiotherapy, immunotherapy, and TKI therapy (29). Results from two multicenter randomized clinical trials showed that advanced NSCLC patients with *KEAP1*/*NFE2L2* mutations had worse clinical outcomes than wild-type patients when treated with immunotherapy and chemotherapy (30). Therefore, the poor prognosis of LMPC patients might be at least partially due to the relatively high frequency of Nrf2 pathway-related aberrations, warranting further research into the efficacy of anti-NRF2 drugs in LMPC.

TMB may also help predict the postoperative prognosis of early-stage NSCLC patients, although its predictive value remains controversial. Previous studies have shown that high TMB is a biomarker of good prognosis in resectable early-stage LADC and NSCLC (31, 32), with patients in the TMB-high group having better DFS and overall survival. However, several studies hold the opposite opinion. A study on Chinese LADC patients reported that high TMB was associated with shorter DFS, and high TMB was more likely to occur in older patients with a smoking history (33). Another study involving 90 patients with early-stage lung cancer reported that high TMB was a poor prognostic factor (34). Besides, higher TMB status conferred a worse implication on OS among patients with non-squamous NSCLC who received platinum-based adjuvant chemotherapy (35). In our study, low TMB was significantly associated with improved DFS in postoperative stage II-III LMPC patients, and there was a high correlation between NPA and TMB. This finding is consistent with previous observations that TMB and NPA are primarily enriched in histologic subtypes of lung cancer with poor prognosis (23). Specifically, we found high TMB correlated significantly with mutations in *TP53*, *KEAP1*, and *SMARCA4*, which have been shown to promote aggressive tumor behavior, and immunosuppressive tumor microenvironments (36–39). These factors may drive early recurrence post-surgery, leading to the observed worse DFS in TMB-high patients in our cohort.

Notably, all genetic profiling in this study was performed on resected tumor tissue. Thus, surgical management was independent of mutation status. However, as liquid biopsy becomes increasingly accurate and available, integrating preoperative genomic data into surgical and perioperative planning may represent a valuable future direction for precision care in early-stage lung cancer.

There were some limitations to this study. Firstly, because the incidence rate of the LMPC subtype is only about 5%, the number of LMPC patients included in this study was relatively small. The small sample size may limit the statistical analyses, impairing the power to detect statistically significant differences in the mutation profiles between the MPC-low and MPC-high groups. Specifically, we acknowledge that the relatively small size of our LMPC cohort may limit the statistical power of multivariate analyses. The loss of statistical significance for SWI/SNF pathway alterations may reflect this limitation rather than a lack of true biological relevance. Further research using larger patient cohorts is needed to fully characterize the LMPC genetic profile. Secondly, as most LADCs are composed of a mixture of multiple histologic subtypes, microdissection of the micropapillary components was not carried out in this study, and the molecular characteristics of the analyzed genes may be affected by the presence of other histologic subtypes. Thirdly, another limitation of our study is that survival data were not available for the reference cohort of 113 LADC patients, which prevented a direct comparison of disease-free survival (DFS) between LMPC and LADC. Future studies integrating molecular and survival data across histologic subtypes will be necessary to fully understand how LMPC differs from conventional lung adenocarcinoma in both biology and clinical behavior. Fourthly, as with many genomic analyses involving multiple comparisons, there is a risk of false-positive (Type I) findings due to the absence of formal adjustment for multiple testing. While we limited our analysis to genes/pathways altered in at least 3 patients, we acknowledge that some associations, particularly those with borderline significance, may not withstand correction for false discovery rate. These findings should therefore be considered exploratory and warrant validation in larger, prospectively designed cohorts. Lastly, all patients included in this study were of East Asian (Chinese) ancestry. Given known ethnic differences in mutational patterns and lung cancer biology, caution should be exercised when generalizing these results to Western or other non-East Asian populations. Future validation in multi-ethnic, geographically diverse cohorts is warranted.

In summary, our study systematically delineated the genetic profile of LMPC and characterized multiple molecular features that differentiate LMPC from other LADC subtypes. Our findings suggest that alterations in *SMARCA4*, *KEAP1*, and components of the SWI/SNF and Nrf2 pathways may be associated with prognosis in stage II-III LMPC. However, these associations were not statistically significant in multivariate analyses and should therefore be validated in larger, multi-center studies. Our study provides a more comprehensive understanding of the molecular characteristics and underlying mechanisms of the poor prognosis of LMPC, aiding in prognostic estimation and treatment decisions in resectable LMPC.

## Data Availability

The sequencing data presented in the study are deposited in the Genome Sequence Archive for Human (GSA-Human) repository (https://ngdc.cncb.ac.cn/gsa-human/), with accession number HRA011575 (https://ngdc.cncb.ac.cn/gsa-human/browse/HRA011575).
