# Supplementary material for: Genomic profiles and prognostic biomarkers of resectable lung adenocarcinoma with a micropapillary component
Source: Front Oncol. 2025 May 29;15:1574817. doi: 10.3389/fonc.2025.1574817 (PMC12158671; doi:10.3389/fonc.2025.1574817)
Supplement: Supplementary file 1 [file DataSheet1.docx]

Supplementary Material

**Supplementary Figure S1**

LADC with at least 5% MPC (n=86)

Inclusion criteria:

- Patients with pathologically confirmed LADC containing at least 5% MPC and negative resection margins;
- Pathological stage I-III;
- Surgical approach involving lobectomy or sublobectomy combined with systematic mediastinal lymph node dissection;
- Patients without significant cardiopulmonary abnormalities or postoperative-specific complications.

32 exclusions:

- Death due to other primary cancers or non-cancer causes (n=6);
- Incomplete clinicopathological records or loss to follow-up (n=17);
- Previous neoadjuvant therapy (n=7);
- Unsuccessful NGS sequencing (n=2).

Total Eligible Patients (n=54)

Figure S1. Flowchart of patient screening in this study. LADC, Lung adenocarcinoma; MPC, micropapillary component.

**Supplementary Figure S2**

Figure S2. The spectrum of oncogenic driver mutations in LMPC patients. The pie charts showed the distribution of main driver mutations (**A**), *EGFR* mutations (**B**), and *KRAS* mutations (**C**) in LMPC patients. LMPC, Lung adenocarcinoma with micropapillary component.

**Supplementary Figure S3**

Figure S3. The prognostic analysis for TNM stage and MPC content in the 54 LMPC patients. (**A**) The Kaplan-Meier curve of DFS stratified by TNM Staging. (**B**) The forest plot of hazard ratios (HRs) for DFS with various MPC cutoffs. (**C**) The Kaplan-Meier curve of DFS in MPC-high and MPC-low patients. MPC, micropapillary component; LMPC, Lung adenocarcinoma with micropapillary component; DFS, disease-free survival; NR, not reached.

**Supplementary Figure S4**

Figure S4. The correlation analysis among individual gene mutations, signaling pathway alterations, and TMB. (A) The correlation matrix of gene mutations and TMB. (B) The relationship between TMB levels and mutational status of individual genes. (C) The correlation matrix of signaling pathways and TMB. (D) The relationship between TMB levels and the alteration of specific signaling pathways. TMB, tumor mutation burden; WT, wild type.

**Supplementary Table S1**

Table S1. Univariate Cox regression analysis of DFS with clinicopathologic characteristics in stage Ⅱ-Ⅲ LMPC patients.

| Characteristics | HR (95% CI) | P value |
| --- | --- | --- |
| **MPC**  High vs Low | 1.31 (0.45-3.84) | 0.619 |
| **Age**  ≥60y vs <60y | 1.28 (0.45-3.66) | 0.644 |
| **Sex**  Female vs Male | 0.52 (0.15-1.86) | 0.306 |
| **Smoking**  Ever vs Never | 2.02 (0.69-5.87) | 0.190 |
| **Lymph node metastasis**  Yes vs No | 1.14 (0.38-3.42) | 0.815 |
| **Pleural invasion**  Yes vs No | 0.25 (0.03-1.91) | 0.147 |
| **Adjuvant therapy**  Yes vs No | 0.69 (0.23-2.08) | 0.512 |
| **Intravascular tumor thrombus**  Yes vs No | 0.90 (0.20-4.08) | 0.890 |

DFS, disease-free survival; LMPC, lung adenocarcinoma with micropapillary component; HR, hazard ratio; CI, confidence interval.

**Supplementary Table S2**

Table S2. Univariate Cox regression analysis of DFS with individual gene or signal pathway alteration in stage Ⅱ-Ⅲ LMPC patients.

|  | **No. of patients (%)** | **HR (95% CI)** | **P value** |
| --- | --- | --- | --- |
| **Gene** |  |  |  |
| *EGFR*  Mut. vs WT | 17 (63.0%) vs 10 (37.0%) | 0.47 (0.16-1.34) | 0.147 |
| *TP53*  Mut. vs WT | 17 (63.0%) vs 10 (37.0%) | 1.68 (0.52-5.36) | 0.379 |
| *LRP1B*  Mut. vs WT | 7 (25.9%) vs 20 (74.1%) | 0.99 (0.32-3.20) | 0.990 |
| *ALK*  Mut. vs WT | 4 (14.8%) vs 23 (85.2%) | 0.78 (0.17-3.48) | 0.740 |
| *KRAS*  Mut. vs WT | 4 (14.8%) vs 23 (85.2%) | 2.87 (0.78-10.50) | 0.098 |
| *NSD1*  Mut. vs WT | 4 (14.8%) vs 23 (85.2%) | 1.93 (0.53-7.06) | 0.312 |
| *PKHD1*  Mut. vs WT | 4 (14.8%) vs 23 (85.2%) | 1.36 (0.38-4.95) | 0.635 |
| *EGFR*  Amp. vs WT | 4 (14.8%) vs 23 (85.2%) | 2.63 (0.71-9.74) | 0.132 |
| *TERT*  Mut. vs WT | 4 (14.8%) vs 23 (85.2%) | 0.26 (0.03-2.06) | 0.173 |
| *SMARCA4*  Mut. vs WT | 4 (14.8%) vs 23 (85.2%) | 4.12 (1.23-13.80) | **0.013** |
| *PIK3CA*  Mut. vs WT | 3 (11.1%) vs 24 (88.9%) | 1.95 (0.54-7.03) | 0.298 |
| *NKX2-1*  Amp. vs WT | 3 (11.1%) vs 24 (88.9%) | 1.32 (0.29-5.97) | 0.717 |
| *SETBP1*  Mut. vs WT | 3 (11.1%) vs 24 (88.9%) | 1.16 (0.25-5.30) | 0.851 |
| *KEAP1*  Mut. vs WT | 3 (11.1%) vs 24 (88.9%) | 2.44 (0.68-8.82) | 0.159 |
| *NOTCH2*  Mut. vs WT | 3 (11.1%) vs 24 (88.9%) | 1.71 (0.38-7.75) | 0.480 |
| *PREX2*  Mut. vs WT | 3 (11.1%) vs 24 (88.9%) | 0.59 (0.08-4.55) | 0.612 |
| *RPTOR*  Mut. vs WT | 3 (11.1%) vs 24 (88.9%) | 0.46 (0.06-3.49) | 0.437 |
| *STK11*  Mut. vs WT | 3 (11.1%) vs 24 (88.9%) | 0.65 (0.08-4.97) | 0.674 |
| **Pathway** |  |  |  |
| RTK_RAS  Alt. vs WT | 26 (96.3%) vs 1 (3.7%) | 0.08 (0.01-0.88) | 0.008 |
| p53  Alt. vs WT | 18 (66.7%) vs 9 (33.3%) | 1.31 (0.41-4.21) | 0.648 |
| PI3K  Alt. vs WT | 11 (40.7%) vs 16 (59.3%) | 0.60 (0.19-1.84) | 0.363 |
| Notch  Alt. vs WT | 7 (25.9%) vs 20 (74.1%) | 1.23 (0.38-3.93) | 0.729 |
| Nrf2  Alt. vs WT | 5 (18.5%) vs 22 (81.5%) | 2.92 (0.97-8.81) | **0.046** |
| Cell Cycle  Alt. vs WT | 4 (14.8%) vs 23 (85.2%) | 1.82 (0.50-6.68) | 0.359 |
| TGFβ  Alt. vs WT | 4 (14.8%) vs 23 (85.2%) | 2.05 (0.57-7.38) | 0.264 |
| Wnt  Alt. vs WT | 4 (14.8%) vs 23 (85.2%) | 1.13 (0.25-5.06) | 0.876 |
| SWI/SNF  Alt. vs WT | 5 (18.5%) vs 22 (81.5%) | 4.89 (1.53-15.60) | **0.003** |
| **Other** |  |  |  |
| TMB  TMB-H vs TMB-L | 10 (37.0%) vs 17 (63.0%) | 3.26 (1.12-9.45) | **0.021** |
| NPA  ≥5 vs < 5 | 3 (11.1%) vs < 24 (88.9%) | 4.14 (1.11-15.40) | **0.021** |

DFS, disease-free survival;LMPC, lung adenocarcinoma with micropapillary component; No., number; HR, hazard ratio; CI, confidence interval; Mut., mutation; WT, wild type; Amp., amplification; TMB, tumor mutation burden; NPA, number of pathway alterations.

**Supplementary Table S3**

| Table S3. Univariate and multivariate Cox regression analysis of DFS with molecular characteristics in stage Ⅱ-Ⅲ LMPC patients. | | | | | |
| --- | --- | --- | --- | --- | --- |
| Molecular characteristics | No. of patients (%) | Univariate analysis | | Multivariate analysis | |
|  |  | HR (95% CI) | P value | HR (95% CI) | P value |
| Nrf2 pathway | 5 (18.5%) | 2.92 (0.97~8.81) | 0.046 | 1.86 (0.56-6.22) | 0.312 |
| SWI/SNF pathway | 5 (18.5%) | 4.89 (1.53~15.60) | 0.003 | 2.89 (0.69-12.19) | 0.147 |
| TMB | 10 (37.0%) | 3.26 (1.12~9.45) | 0.021 | 1.65 (0.41-6.69) | 0.480 |

DFS, disease-free survival; LMPC, lung adenocarcinoma with micropapillary component; No., Number; TMB, tumor mutation burden; HR, hazard ratio; CI, confidence interval.
